# Evaluation of an experiment to increase availability of healthier snack foods in vending machines situated within English sports facilities

**DOI:** 10.1017/S1368980023002069

**Published:** 2023-12

**Authors:** Charlotte EL Evans, Stuart Worth, Rachel White, Emma K Strachan

**Affiliations:** 1 School of Food Science and Nutrition, University of Leeds, Leeds, LS2 9JT, UK; 2 Wilkes Group, Ossett, UK; 3 Health Improvement and Disparities, Department of Health and Social Care, London, UK; 4 Leeds City Council, Leeds, UK

**Keywords:** Vending machines, Food purchasing, Nutritional quality, Community food environment, Evaluation

## Abstract

**Objective:**

To evaluate the impact of increased availability of healthier options on purchasing of different types of vending snack products sold in English leisure (sports) centres.

**Design:**

An evaluation of an intervention using pre-post methods and interrupted time series analysis. Products within the vending machines were altered over three phases to increase the availability of healthier options, using agreed nutrition criteria – Government Buying Standards for Food and Catering Services (GBSF) for England – as a guide, as well as product availability. The primary outcome was the change in mean weekly purchased energy between the first and third phase. Secondary outcomes included changes by phase and by week in weekly number of purchases, fats, sugars and salt for all products combined and by individual product categories.

**Setting:**

Fifteen sports centres in the city of Leeds, West Yorkshire, UK.

**Participants:**

Snack products sold in eighteen vending machines.

**Results:**

Energy purchased reduced from baseline to phase 2, for all product categories combined, by 47·25 MJ (95 % CI (−61·22, −33·27)) per machine and by 279 kJ, (95 % CI (−325, −266)) per product unit. There were reductions in most nutrients purchased in all individual product categories except chocolate confectionery. Nutrients per product unit decreased for all product categories except saturated fat in chocolate confectionery. Minimal underlying trends in the baseline phase were identified, indicating changes in outcomes were likely to be due to the intervention.

**Conclusions:**

Introducing standards to increase availability of healthier snack products in vending machines is feasible without substantially affecting sales.

Obesity has increased globally by 10-fold in the past 40 years in part due to poor diet^(1)^. A review of eating behaviours and excess body weight, in general populations of adults and children, demonstrated positive associations between snacking and risk of obesity, likely due to excess energy intake^(2)^. Unhealthy food environments influence an individual’s ability to make healthier dietary choices^(3,4)^, and foods that are purchased outside the home are typically less healthy^(5)^. The WHO has highlighted that action must be taken against unhealthy diets to reduce premature deaths by non-communicable disease by a third by 2030^(1)^ including improvements in food environments^(6)^. In England, where this study is based, improving access to healthier food in public spaces is a priority action^(3)^, as outlined in the government’s childhood obesity plan for England (health is devolved in the UK)^(7)^.

Vending machines are common sources of snack foods in many countries with an estimated half a million vending machines in the UK, equivalent to one vending machine per fifty-five people, vending more than 7 billion items per year^(8)^. Density of vending machines in the USA is similar^(9)^. US data report that 4 % of American adults purchase food from a vending machine on any given day in different settings, including recreational centres, with purchases usually comprised of sugary drinks, salty snacks or confectionery^(10)^. Although comparable data from the UK are not currently available, it is likely to be similar due to the equally high density of vending machines. Furthermore, the UK contains more machines than anywhere else in Europe with about one in eight European machines located in the UK^(8)^.

Traditionally, leisure facilities in high-income countries rarely provide a supportive environment for healthy food choices^(11)^ with the majority of snack foods and drinks in sports facilities typically classified as unhealthy whether purchased from vending machines or cafes^(12,13)^. A systematic review of interventions to improve the quality of foods offered in vending machines confirmed that vending machines are generally associated with pre-packaged snack foods typically high in fats, sugars and salt^(14)^. In the UK, overall vended snack consumption is falling, although the vast majority of sports facilities in the UK still have a vending machine within their environment^(15,16)^. Sports facilities provide an opportunity to set an example in providing a healthier food environment as well as encouraging children to be more active.

Healthier public sector catering, which includes vending, has a key part to play to improve food environments, alongside national interventions to encourage healthier choices. Government Buying Standards for Food and Catering Services (GBSF) in England aim to ensure healthier food and drink options are available across public sector settings, including central government departments and NHS hospitals^(17)^. Local contracts, which include criteria for healthier food and drinks, are shown to improve food environments in community spaces highlighting this as a successful approach^(18)^. Leeds City Council (LCC) was an early adopter of the Food Active Healthy Weight Declaration^(19)^, one commitment being a declaration to make healthier food and drinks more available, accessible and affordable. Previous action on vending machines in Leeds within the hospital setting, which provided learning for this project, evaluated changes in product availability and placement on purchasing behaviour^(3)^. In line with previous research^(14,20)^, this project reported product availability as having the most impact. However, to ensure financial viability of food providers, sales need to be maintained. An Australian trial^(21)^ focusing on altering the availability of sugary drinks resulted in reduced purchases of products classified as less healthy, but this was only partially offset with increases in healthier products leading to reductions in purchases overall. Removing popular products completely could also drive customers to other food outlets and result in less healthy food choices overall^(16)^; therefore, product substitutions need to be carefully considered.

The aim of this study is to evaluate the impact of an intervention in eighteen sports facility vending machines on the purchases of healthier vended snacks between September 2018 and May 2019. Robust evaluation methods are being developed to evaluate interventions in a range of food environments that are valid but cheaper and quicker than randomised controlled trials, including interrupted time series (ITS) analysis. The main aim of this intervention was to transform the offerings in two intervention phases to encourage healthier vended snack choices and compare with the baseline pre-intervention period. The primary outcome was change in mean weekly purchased energy between baseline and phase 2 overall for the pre-post analysis. Secondary outcomes included changes by phase and by week in weekly number of purchases, total and saturated fats, sugars and salt purchased for all products combined and by each of the four individual product categories (savoury snacks, chocolate confectionery, sweet confectionery and other sweet products).

## Methods

The trial was coordinated by LCC and Wilkes Group (a large vending supplier and existing Leeds City Council provider), with technical support from (then) Public Health England (PHE) and evaluated by the University of Leeds. The study protocol (unpublished) built on learning from the Leeds Teaching Hospitals NHS Trusts project^(22)^.

The trial design included three phases, spanning 8 months, to improve the availability of healthier snacks in vending machines. All vending machines were included in the intervention, and there were no control machines. The eighteen sports centre vending machines in fifteen sports facilities were selected based on the participating vending company that supplied the vending products in all the machines. All vending machines in Leeds sports centres supplied by the participating vending company were included in the study. One large centre included four vending machines in separate buildings, but the remaining sports centres all contained one machine. The sports centres were all public facilities of varying sizes that were free to enter. Customers pay for swimming sessions or sports classes at point of use. The facilities were located in a variety of neighbourhoods including lower and higher income areas. Purchases made from vending machines in the sports facilities constitute purchases by visitors, including children, as well as staff working in the leisure centre facilities.

The trial took place between September 2018 and March 2019 and consisted of the following phases: baseline for 8·5 weeks from 3 September until 31 October, phase one for 10 weeks from 1 November 2018 until 13 January 2019 and phase 2 for 8 weeks from 14 January 2019 to 13 March 2019. During the second intervention phase, it was agreed to continue the intervention into an exploratory third phase for 11 weeks from 14 March 2019 to 31 May 2019, but the results of this third intervention phase are not reported here.

The vending machine supplier supplied the vending snacks for all phases and collected weekly purchased data for individual products from each machine. The intervention phases involved taking a stepped approach, changing the products within the vending machine with the aim of increasing availability of healthier products. Products within the machines were changed in line with planograms based on nutrition criteria (energy thresholds and portion size) developed and agreed for each phase in collaboration with LCC and PHE. The nutrition criteria were informed by best practice criteria within the GBSF in place at the time, as well as PHE sugar reduction and energy guidelines^(23)^ and products available to the vending company. Products were grouped into four categories: savoury snacks (which included crisps, lentil chips, savoury popcorn and savoury crackers), chocolate confectionery (which included chocolate bars), sweet confectionery (which included fruit and mint flavoured non-chocolate confectionery) and other sweet snacks (which included cakes and biscuits/cookies and cereal bars). No machines providing drinks were included in this study.

Like-for-like changes within product categories were made within each position in the vending machines to ensure product type did not vary. For example, a savoury snack product was replaced by an alternative savoury snack product rather than chocolate confectionery. During the baseline phase, no changes to existing products were made. In the two intervention phases, non-compliant products were removed from the baseline vending offer and a swap made using a compliant product from the same product category. During phase one, savoury snacks were required to be 30 g or less to be included. Sweet snacks and confectionery were required to be no more than 250 kcal. Additionally, other sweet snacks not covered by the GBSF criteria were required not to exceed 325 kcal, reflecting sugar reduction and energy guidelines for these products. For phase 2, savoury snacks were required to be 30 g or lower, chocolate and sweet confectionery were required to be 230 kcal and 200 kcal or lower, respectively, and other sweet snacks were required to be 250 kcal or lower. All sales from each vending machine were recorded weekly by the vending company. Nutritional information including energy, saturated fat, sugars and salt were collated by LCC and shared with UoL. Changes to provision were not communicated to consumers, through a marketing strategy, for example, in either phase 1 or 2 and prices did not change.

### Ethics

Ethics approval was not required as the study took an environmental approach, and no data were collected from individuals.

### Data analysis

Prior to analysis, the sales data from Wilkes Group were checked and cleaned for errors or inconsistencies. For example, missing or misreported data were rectified where possible by averaging over two time points if data for 1 week had all been reported for the following week. Missing nutritional information was completed using products found on supermarket or supplier websites in order to provide complete nutrient data for all products.

Values of main outcomes were calculated per phase and reported as mean values per machine. Values for units sold, energy, total and saturated fat, sugars, salt and total weight of products were calculated for each phase for all products and by the four individual product categories. Values were converted to weekly figures by dividing total values by the number of weeks in each phase. Mean energy (kJ and kcal), total fat, saturated fat, salt, sugars and weight (all grams) per product unit sold were also calculated for each phase for all products and by product category to take account of any differences in sales.

Two complementary approaches were used to evaluate the success of the programme based on the primary outcomes of changes in energy and nutrients purchased from vending machines. Firstly, regression analysis was carried out comparing phase 2 with the baseline phase. Secondly, ITS analysis was applied to the data, which takes into account any underlying trends during the baseline phase.

To determine differences for energy and nutrients in phase 2 compared with baseline values, multilevel regression analysis was carried out. Sales patterns for different products and product categories may have varied by machine due to differences in footfall and usage patterns between the sports centres. Therefore, in order to account for this, two-level regression models were used where possible with one level being an individual product and the second level being an individual machine. The outcome variable for each regression model was energy, nutrient or sales with phase as the main predictor variable, for all product units and product categories. Mean differences from baseline to intervention phase (negative values indicate a reduction in values in the intervention phase) and 95 % CI were reported for each model. The analysis was repeated to provide results per individual product unit. The total energy and nutrient value was divided by the number of product units sold, for all products and for each product category.

Secondly, ITS analysis was carried out to assess any underlying trends during the baseline phase. Subsequent changes during the intervention phases are then adjusted for any underlying trends detected. For example, independent of this intervention there may have been changes in energy purchased from snacks due to national changes in reformulation of snack products due to national policies to reduce sugars and energy.

The ITS analysis was carried out on all vending machines combined for each of the main primary outcomes, energy, total and saturated fats, sugars, salt and sales, using segmented regression with three phases (baseline, phase 1 and phase 2) on weekly purchasing data. The analysis was carried out with STATA 14 following the ‘itsa’ programme^(24)^. The model measured the difference between the existing model with the data collected from the study and the counterfactual which is what would have occurred if the intervention phases had not been implemented. The fitted model used was the Newey–West estimation method testing for lags up to 10. The fitted model assumed an autoregressive correlation lag 2 as lags were not significant after 1 for any model. More details on the mathematical models and the assumptions made using ITS analysis are provided by Linden^(24)^. The analyses tested the differences in level (of purchases of energy and nutrients) between phases for each outcome and for significant trends (increases or decreases) within phases for all product categories combined. The process was repeated to look at purchased energy, total and saturated fat, sugars, salt and weight of individual units sold. Although we did not have footfall data to adjust for particular events taking place which could affect sales, we did adjust for holiday periods which also have an impact on leisure centre use and therefore would be likely to affect vending sales.

A power calculation undertaken retrospectively at the end of the study based on the standard deviation for energy of snacks indicated that information from 500 sales would be sufficient to detect differences in the region of 10 % difference with 90 % power, and we collected data on approximately 3000 products weekly.

## Results

Results of sales are discussed first followed by results for energy and nutrients.

### Sales

**Fig. 1:**
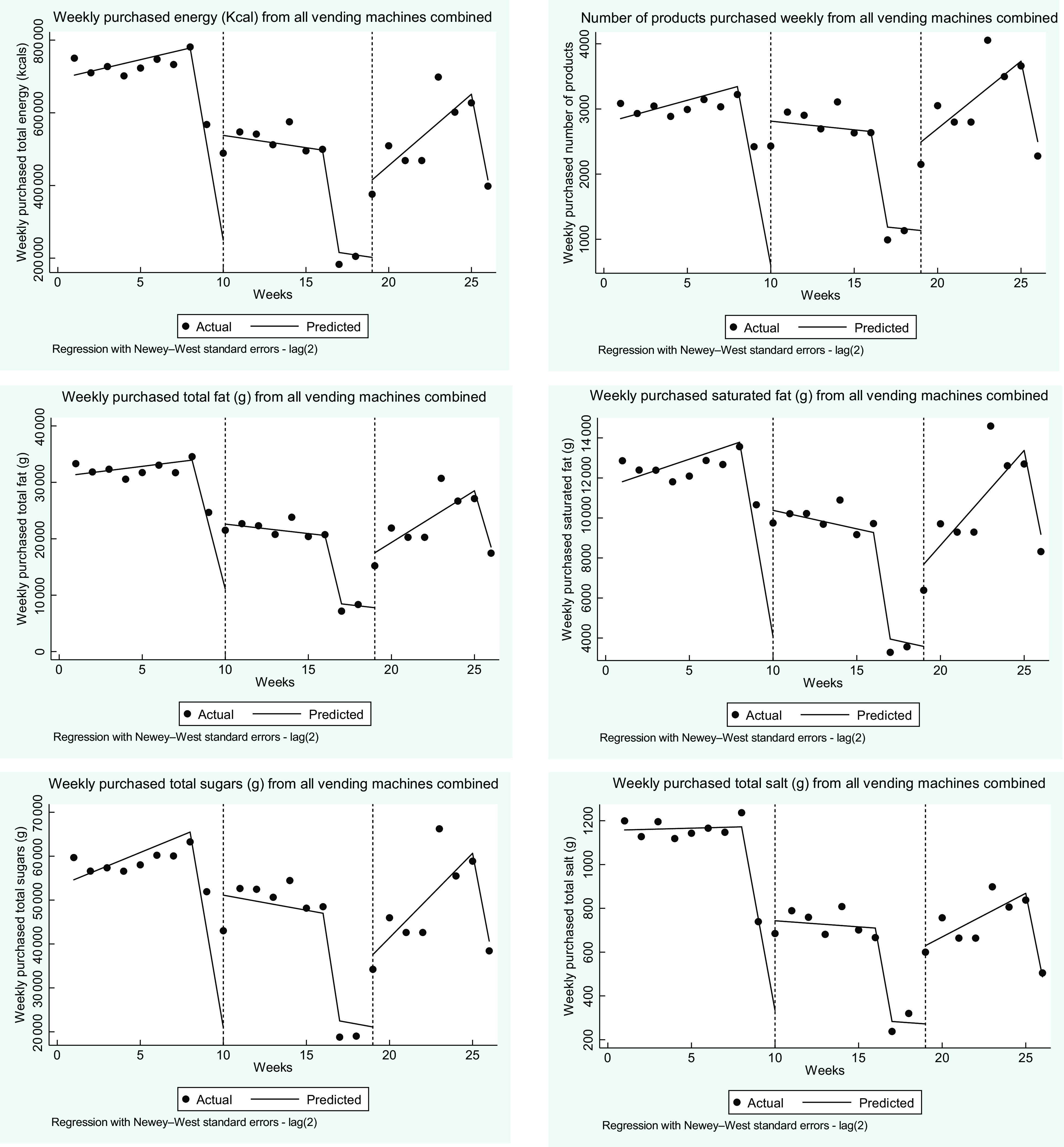
Time series analysis for all purchased products during baseline (weeks 1 to 9), phase 1 (weeks 10 to 18) and phase 2 (weeks 19 to 26). Vertical dotted lines represent the start of a new phase. Results (black dots) show actual weekly values for number of sales, energy, total fat, saturated fat, total sugar and salt purchased from all vending machines combined. Lines are predicted values adjusted for school holiday weeks (weeks 9, 17, 18 and 26)

For all product categories, combined sales were similar in phase 2 compared with baseline after dipping in phase 1 (Table 1). The mean number of product units sold per week in each machine was 171 items during the baseline phase and 178 during phase 2 (Table 1) for all product categories combined. There were some differences by product category; in phase 2, sales of savoury snacks and sweet confectionery recovered to baseline levels and chocolate confectionery significantly increased above baseline levels, whereas other sweet products significantly decreased. ITS analysis of data within phases indicated that sales increased during the baseline phase, followed by a decrease during phase 1 and then increased again during phase 2 (Fig. 1) which could be due to seasonality or additional unknown factors.

**Table 1:** Mean weekly energy, nutrients and units purchased for total products and by product category per vending machine for each phase

Weekly mean per vending machine	Baseline	Phase 1	Difference between baseline and phase 1	95 % CI	*P* value baseline and phase 1	Phase 2	Difference between baseline and phase 2	95 % CI	*P* value baseline and phase 2
Total products									
Product units sold	171	128	−42·9	−57·7, 28·1	<0·001	178	6·8	−6·1, 19·6	0·282
Energy (MJ)	172·79	101·14	−71·64	−89·94, −53·35	<0·001	125·54	47·25	61·22, −33·27	<0·001
Energy (kcals)	41 297	24 174	−17 123	−21 495, −12 750	<0·001	30 005	−11 292	−14 633, −7951	<0·001
Total fat (g)	1821	1001	−819	−1017, −622	<0·001	1319	−502	−647, −356	<0·001
Saturated fat (g)	709	455	−253	325, −182	<0·001	617	−92	−148, −36	0·003
Total Sugars (g)	3333	2312	−1020	−1338, −703	<0·001	2731	−601	−853, −349	<0·001
Salt (g)	65·2	33·8	−31·5	−39·6, −23·3	<0·001	41·9	−23·3	−30·2, −16·5	<0·001
Savoury snacks									
Product units sold	57·0	39·5	−17·5	−23·0, −12·0	<0·001	55·6	−1·4	−6·1, 3·3	0·542
Energy (MJ)	46·84	18·90	−27·94	−34·84, 21·05	<0·001	26·18	−20·66	−26·06, −15·27	<0·001
Energy (kcals)	11 195	4517	−6678	−8326, −5030	<0·001	6256	−4939	−6229, −3649	<0·001
Total fat (g)	600	209	−392	−485, −299	<0·001	290	−311	−386, −236	<0·001
Saturated fat (g)	83·4	20·7	−62·7	−78·9, −46·6	<0·001	26·8	−56·7	−71·5, −41·9	<0·001
Total Sugars (g)	93·1	34·3	−58·6	−73·9, −43·4	<0·001	46·8	−46·3	−58·6, −34·0	<0·001
Salt (g)	35·6	20·1	−15·5	−19·7, −11·3	<0·001	28·1	−7·5	−11·1, −3·8	<0·001
Chocolate confectionery									
Product units sold	52·1	50·4	−1·6	−9·1, 5·8	0·648	76·7	24·6	14·0, 35·3	<0·001
Energy (MJ)	49·91	48·31	−1·60	−8·77, 5·56	0·643	67·42	17·51	8·16, 26·85	<0·001
Energy (kcals)	11 929	11 546	−383	−2096, 1329	0·643	16 113	4184	1950, 6418	<0·001
Total fat (g)	608	589	−18	−105, 68	0·657	863	255	137, 373	<0·001
Saturated fat (g)	345	335	−9·2	−58·4, 39·9	0·696	506	161	92·9, 230	<0·001
Total Sugars (g)	1241	1206	−35·1	−218, 148	0·691	1713	472	237, 707	<0·001
Salt (g)	7·0	6·8	−0·16	−1·13, 0·81	0·733	8·6	1·62	0·38, 2·86	0·013
Other confectionery (includes cakes and biscuits)									
Product units sold	35·4	17·3	−18·1	−23·1, −13·0	<0·001	19·5	−15·9	−20·6, −11·2	<0·001
Energy (MJ)	51·07	17·45	−33·61	−42·93, −24·29	<0·001	14·36	−36·71	−47·14, −26·27	<0·001
Energy (kcals)	12 205	4171	−8034	−10,261, −5806	<0·001	3432	−8773	−11,267, −6279	<0·001
Total fat (g)	572	170	−403	−515, −291	<0·001	137	−435	−558, −312	<0·001
Saturated fat (g)	261	82·3	−179	−230, −128	<0·001	66·3	−195	−251, −139	<0·001
Total Sugars (g)	928	307	−620	−795, −446	<0·001	228	−699	−900, −499	<0·001
Salt (g)	21·3	5·8	−15·6	−20·4, −10·7	<0·001	3·3	−18·0	−23·8, −12·2	<0·001
Sweet confectionery									
Product units sold	26·8	21·1	−5·7	−8·6, −2·8	0·001	26·2	−0·6	−2·9, 1·7	0·599
Energy (MJ)	24·97	16·48	−8·48	−11·82, −5·15	<0·001	17·59	−7·38	−10·31, −4·46	<0·001
Energy (kcals)	5968	3940	−2027	−2824, −1231	<0·001	4204	−1764	−2463, −1065	<0·001
Total fat (g)	39·8	33·7	−6·2	−10·5, −1·9	0·008	29·5	−10·3	−14·4, −6·2	<0·001
Saturated fat (g)	19·2	17·0	−2·3	−4·5, −.1	0·045	17·9	−1·3	−3·4, 0·8	0·202
Total Sugars (g)	1071	765	−306	−431, −182	<0·001	743	−328	−442, −214	<0·001
Salt (g)	1·3	1·1	−0·25	−0·45, −.05	0·019	1·8	.49	0·18, 0·79	0·004

### Energy and nutrients

Overall mean energy from all products purchased weekly per machine reduced from 41 297 kcals during the baseline phase to 30 005 kcal in phase 2 (*P* < 0·001) (see Table 1). There were differences by product category: energy from savoury products reduced from 11 195 kcal at baseline to 6 256 kcal in phase 2 (*P* < 0·001) and energy from sweet confectionery reduced from 5 968 kcal at baseline to 4 204 kcal at phase 2 (*P* < 0·001). The largest reduction in energy was for other sweet products such as cakes and biscuits which substantially reduced from 12 205 kcal at baseline to 3 432 kcal in phase 2 (*P* < 0·001). However, purchased energy increased for the product category chocolate confectionery from 11 929 kcal at baseline to 16 113 kcal in phase 2 (*P* < 0·001). Nutrients from all products purchased including total fat, saturated fat, sugars and salt showed similar patterns (see Table 1). The only differences between nutrients being that the reduction in saturated fat for sweet confectionery was smaller (see Table 1) as would be expected from a product already low in saturated fat. For all product categories, ITS analysis of differences between and within phases indicated that purchases of energy, total fat and saturated fats and salt were stable during the baseline phase indicating minimal underlying trends due to factors external to the intervention (Table 2). There were substantial reductions in levels of energy and all nutrients from baseline to phase 1 and a further reduction in energy, but not individual nutrients, between phase 1 and phase 2 (Table 2). There were trends within the intervention phases with energy and nutrients generally decreasing during phase 1 and increasing during phase 2 (Table 2) which could be due to seasonality or other factors unrelated to the intervention.

**Table 2:** Results on energy, nutrients and unit sales from interrupted time series analysis for all products in all eighteen vending machines combined and per unit sold

Nutrient weekly levels	Time	95 % CI	Level 1	95 % CI	Level 1 trend	95 % CI	Level 2	95 % CI	Level 2 trend	95 % CI
For all machines combined										
Energy (MJ)	44·61	−0·51, 89·74	−1094·8**	−1451·7, −737·9	−72·85**	−117·5, −28·25	−258·2*	−511·4, −4·97	192·6**	138·8, 246·3
Energy (kcals)	10 663	−121, 21 448	−261 669**	−346972, −176366	−17 412**	−28073, −6752	−61 708*	−122,228, −1188	46 028**	33 185, 58 870
Total fat (g)	367	−111, 844	−12 069**	−15,182, −8957	−703**	−1189, −217	−2069	−4852, 715	2170**	1583, 2756
Saturated fat (g)	282	−11·8, 575·4	−3965**	−5939, −1991	−466**	−793, −139	−1034	−2831, 763	1133**	757, 1509
Total sugars (g)	1555*	159, 2950	−17 492**	−30141, −4843	−2241**	−3739, −742	−7363	−15683, 957	4532**	2840, 6224
Salt (g)	2·0	−8·1, 12·1	−433**	−534, −332	−7·6	−19·7, 4·6	−63·9	−166, 38·5	45·4**	30·4, 60·4
Unit sales	70·2*	1·5, 139	−671*	−1275, −67	−96·7*	−168·6, −24·8	−82·2	−547·1, 382·6	233·3**	132·8, 333·9
Per product unit sold										
Energy (kJ)	−2·1	−4·18, 0·4	1904**	−225, −156	−2·9	−7·5, 2·1	−56**	−95, −17	7·5*	0·8, 13·8
Energy (kcals)	−0·5	−1·0, 0·1	−45·5**	−53·7, −37·2	−0·7	−1·8, 0·5	−13·4**	−22·8, −4·1	1·8*	0·2, 3·3
Total fat (g)	−0·04**	−0·08, −0·01	−2·21**	−2·83, −1·59	−0·05	−0·16, 0·05	−0·23	−0·77, 0·30	0·19**	0·09, 0·29
Saturated fat (g)	0·02	−0·13, 0·05	−0·50**	−0·79, −0·21	−0·08*	−0·15, −0·01	−0·15	−0·58, 0·27	0·15**	0·09, 0·21
Total sugars (g)	0·14*	0·02, 0·25	−2·04**	−3·20, −0·87	−0·21	−0·44, 0·01	−2·20*	−3·96, −0·44	0·20*	0·01, 0·39
Salt (g)	−0·005*	−0·010, −0·001	−0·086**	−0·125, −0·047	0·006	0, 0·012	−0·011	−0·048, 0·025	−0·005	−0·014, 0·003
Weight (g)	−0·10	−0·25, 0·05	−10·5**	−12·2, −8·7	−0·07	−0·28, 0·13	−3·00**	−4·79, −1·12	0·22	−0·10, 0·54

Time indicates the increase or decrease per week during baseline, level 1 indicates the change in energy, nutrient or sales from baseline to phase 1, level 1 trend indicates the increase or decrease per week in phase 1, level 2 indicates the change between phase 1 and phase 2, and level 2 trend indicates the increase or decrease per week during phase 2. Statistically significant changes are indicated by *(*p* < 0.05) or **(p < 0.01).

In terms of the energy and nutrient profile per product unit sold, for all product categories combined, mean energy reduced significantly from 240 kcal at baseline to 169 kcal at phase 2 (Table 3). For individual product categories, energy reduced for all categories (*P* < 0·001 for all categories). For savoury products energy reduced from 197 kcal to 114 kcal from baseline to phase 2, for other sweet products from 341 kcal to 175 kcal, for sweet confectionery from 219 kcal to 158 kcal and for chocolate confectionery from 229 kcal to 210 kcal. Trends for total fat, saturated fat, sugars and salt were generally similar for individual product categories, reducing for all nutrients between baseline and phase 2 except for saturated fat in chocolate confectionery and salt in sweet confectionery which increased slightly (albeit from a low base for the latter case).

**Table 3:** Mean energy and nutrient content per product unit sold for total products and by product category for each phase

Product mean	Baseline	Phase 1	Change from baseline to phase 1	95 % CI	*P* value from baseline to phase 1	Phase 2	Change from baseline to phase 2	95 % CI	*P* value from baseline to phase 2
All products: Per unit sold									
Energy (kJ)	1004	791	−213	−240, −185	<0·001	707	−295	−325, −266	<0·001
Energy (kcals)	240	189	−50·8	−57·4, −44·2	<0·001	169	−70·6	−77·6, −63·6	<0·001
Total fat (g)	10·7	7·9	−2·8	−3·1, −2·4	<0·001	7·5	−3·2	−3·5, −2·9	<0·001
Saturated fat (g)	4·2	3·6	−0·57	−0·72, −0·42	<0·001	3·5	−0·66	−0·80, −0·51	<0·001
Total sugars (g)	19·4	18·0	−1·4	−2·1, −0·7	<0·001	15·4	−3·96	−4·7, −3·2	<0·001
Salt (g)	0·38	0·26	−0·11	−0·13, −0·09	<0·001	0·24	−0·14	−0·16, −0·12	<0·001
Weight (g)	51·2	40·0	−11·3	−12·9, −9·7	<0·001	35·6	−15·6	−17·4, −13·9	<0·001
Savoury snacks: Per unit sold									
Energy (kJ)	824	485	−339	−352, −325	<0·001	477	−348	−371, −324	<0·001
Energy (kcals)	197	116	−81·0	−84·2, −77·7	<0·001	114	−83·1	−88·7, −77·5	<0·001
Total fat (g)	10·6	5·3	−5·26	−5·58, −4·93	<0·001	5·2	−5·36	−5·85, −4·87	<0·001
Saturated fat (g)	1·48	0·54	−0·94	−1·04, −0·83	<0·001	0·48	−1·00	−1·10, −0·89	<0·001
Total sugars (g)	1·63	0·89	−0·74	−0·82, −0·67	<0·001	0·85	−0·78	−0·83, −0·73	<0·001
Salt (g)	0·63	0·51	−0·12	−0·13, −0·10	<0·001	0·51	−0·12	−0·14, −0·10	<0·001
Weight (g)	38·9	24·1	−14·8	−15·5, −14·1	<0·001	23·7	−15·2	−16·16, −14·2	<0·001

Weekly mean	Baseline	Phase 1	Change from baseline to phase 1	95 % CI	*P* value from baseline to phase 1	Phase 2	Change from baseline to phase 2	95 % CI	*P* value from baseline to phase 2
Chocolate confectionery: per unit sold									
Energy (kJ)	958	958	2	−2, 5	0·398	879	−79	−85, −73	<0·001
Energy (kcals)	229	229	0·4	−0·5, 1·3	0·398	210	−18·9	−20·3, −17·5	<0·001
Total fat (g)	11·7	11·7	0·02	−0·07, 0·10	0·729	11·2	−0·44	−0·54, −0·34	<0·001
Saturated fat (g)	6·6	6·6	0·02	−0·05, 0·10	0·538	6·6	−0·03	−0·13, 0·08	0·603
Total sugars (g)	23·8	24·0	0·17	−0·16, 0·46	0·223	22·4	−1·47	−1·89, −1·06	<0·001
Salt (g)	0·13	0·14	0·003	−0·001, 0·006	0·103	0·11	−0·021	−0·025, −0·017	<0·001
Weight (g)	45·0	45·2	0·12	−0·15, 0·40	0·360	40·3	−4·75	−5·14, −4·36	<0·001
Other sweet products (including cakes and biscuits): per unit sold									
Energy (kJ)	1427	996	−429	−493, −365	<0·001	732	−695	−738, −651	<0·001
Energy (kcals)	341	238	−102·5	−117·8, −87·2	<0·001	175	−166·1	−176·5, −155·6	<0·001
Total fat (g)	16·0	9·7	−6·32	−7·18, −5·45	<0·001	6·9	−9·06	−9·73, −8·39	<0·001
Saturated fat (g)	7·2	4·6	−2·64	−3·04, −2·24	<0·000	3·3	−3·93	−4·26, −3·59	<0·001
Total sugars (g)	25·8	17·4	−8·44	−9·82, −7·07	<0·001	11·6	−14·21	−15·11, −13·31	<0·001
Salt (g)	0·58	0·33	−0·250	−0·306, −0·195	<0·001	0·18	−0·403	−0·462, −0·344	<0·001
Weight (g)	75·2	50·9	−24·3	−27·6, −21·0	<0·001	38·1	−37·0	−39·4, −34·7	<0·001
Sweet confectionery: per unit sold									
Energy (kJ)	916	778	−138	−196, −79	<0·001	661	−253	−312, −194	<0·001
Energy (kcals)	219	186	−32·9	−46·9, −18·8	<0·001	158	−60·5	−74·6, −46·4	<0·001
Total fat (g)	1·5	1·5	0·05	−0·05, 0·15	0·288	1·0	−0·43	−0·53, −0·33	<0·001
Saturated fat (g)	0·7	0·8	0·07	0·03, 0·11	0·003	0·6	−0·05	−0·11, −0·001	0·047
Total sugars (g)	39·7	35·9	−3·81	−5·66, −1·97	<0·001	27·8	−11·87	−13·75, −9·99	<0·001
Salt (g)	0·05	0·05	0·002	−0·003, 0·008	0·334	0·06	0·019	0·011, 0·026	<0·001
Weight (g)	58·3	48·2	−10·1	−14·4, −5·8	<0·001	44·8	−13·5	−17·6, −9·3	<0·001

**Fig. 2:**
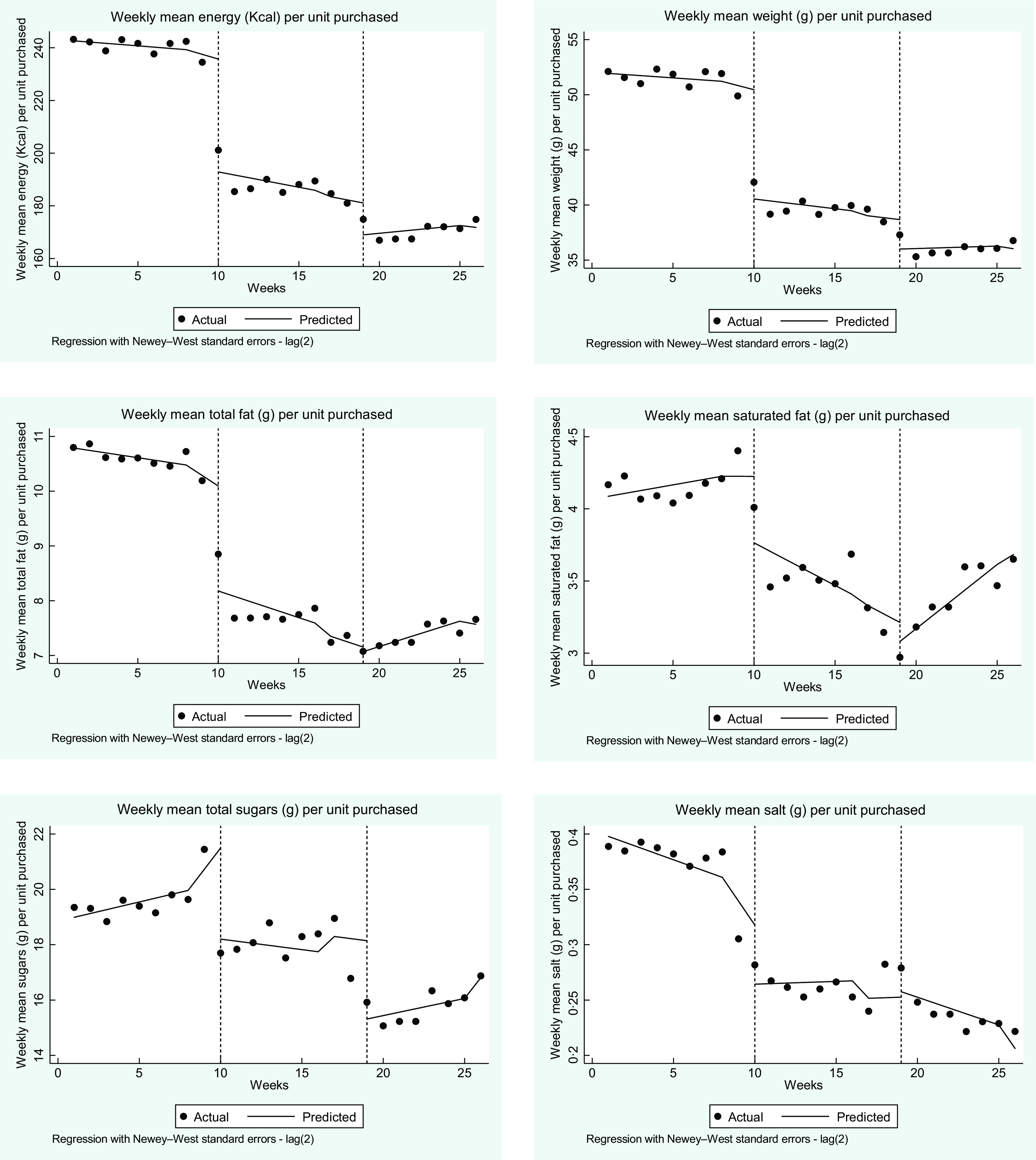
Time series analysis results per item purchased during baseline (weeks 1 to 9), phase 1 (weeks 10 to 18) and phase 2 (weeks 19 to 26). Vertical dotted lines represent the start of a new phase. Results (black dots) show actual weekly values for number of sales, energy, total fat, saturated fat, total sugar and salt purchased from all vending machines combined. Lines are predicted values adjusted for school holiday weeks (weeks 9, 17, 18 and 26)

ITS analysis of trends between and within phases for individual product units indicated that during the baseline phase energy content of products did not change, similar to total energy sold; however, total fat and salt decreased during the baseline phase (Fig. 2). There were substantial differences between baseline and phase 1 in energy and all nutrients per unit as well as the weight in grams of each product unit. Additional changes between phase 1 and phase 2 included further reductions in energy per unit as well as further reductions in total sugars and weight of products. There was a trend for some nutrients per unit sold to moderately increase during phase 2 including saturated fats and sugars (Table 2), driven by the increase in proportion of purchases being chocolate confectionery.

## Discussion

This intervention aimed to improve availability of healthier products in vending machines in sports facilities in a large city in the UK and thereby increase purchasing of healthier snack foods. Overall, the intervention succeeded in maintaining sales while reducing levels of energy, total and saturated fats, sugars and salt purchased from vending machines by around 20 to 30 % between baseline and phase 2. Reductions in energy, fats, sugars and salt purchased were also seen for most of the individual product categories although the impact varied. However, there was an increase in saturated fat purchased from chocolate confectionery products, which was partly due to the increase in the proportion of purchases being made from this product category at phase 2 and also because the majority of chocolate confectionery products met the nutrition criteria at baseline and therefore fewer changes were made in this product category. In addition, the data suggest there were some shifts in sales between product categories. The substantial reduction in sales of ‘other sweet products’ such as cakes and biscuits, which reduced from 20 % to 15 % of total sales between baseline and phase 2, compared to chocolate confectionery which increased from 30 to 39 % of total sales, may have been due to the relative lack of choice of like-for-like products for cakes and biscuits during the intervention leading to customers crossing over to purchasing chocolate confectionery. Implementation of the criteria resulted in substitutions within a category to meet energy thresholds which may not have been as popular (e.g. cookies for rice cakes). Sales of savoury products remained stable, contributing to 33 % of the total number of sales at baseline indicating that customers may be less likely to cross over from savoury to sweet or vice versa.

In this study, the healthier snacks are either smaller in portion size or, less commonly, lower in energy density (energy per gram) or a mixture of both approaches. Previous work has highlighted the impact of different strategies to improve healthy food choices. High accessibility is an important driver of vending machine sales^(25)^. Fewer vending machines mean reduced access to less healthy snack products, but strategies such as increasing the proportion of healthier products within vending machines as well as in other food outlets such as concessions are also reported to be effective and more so than promotional methods and providing information^(16,22,26,27)^. Importantly, recreational centres in more deprived areas may be less likely to implement healthier food environments with more availability of healthier options^(28)^ if new standards are not implemented in all regions. Strategies to increase availability of healthier options are therefore more likely to be more successful if incorporated into mandated nutrition policies to ensure a level playing field. Increasing price of energy-dense sugary foods and reducing price of healthier foods may also have an impact on food choice^(29,30)^, but this was not tested here as prices remained the same throughout.

The snack foods in the vending machines in this study did not include any fresh foods such as fruit. This was due to a lack of suitable vending and storing conditions in the machines. However, this project has shown that healthier options lower in energy, fats, sugars and salt can be provided that are acceptable to both consumers and business interests. Much of the improvement was achieved through reductions in portion size (including energy thresholds), rather than energy density of products which remained stable at approximately 4·6 kcal/g overall (data not shown). Interestingly, saturated fat, per unit sold, for chocolate confectionery remained the same despite reductions in energy. Considering nutritional profiles alongside reductions in portion size would result in further improvements in vending machine contents.

Public reaction to increasing the proportion of healthier options in vending machines is generally reported to be positive^(31)^, demonstrating opportunities for effective health interventions and indeed there were no reported strong negative reactions to this intervention in terms of sales. Although sales dipped in phase 1, potentially due to seasonal effects (phase 1 straddled the Christmas period) as well as due to potential customer dissatisfaction (although no data on satisfaction were collected), sales recovered in phase 2. Eliminating less healthy foods and drinks high in fats, salt and/or sugars completely, on the other hand, is a drastic intervention approach which has been associated with low acceptability among consumers^(16)^. A more holistic and flexible approach which ensures customers have access to healthier products in food outlets as well as vending machines in community food environments is more likely to be acceptable as well as successful in meeting the needs of multiple stakeholders.

There were strengths to the study. This trial involved working collaboratively with an existing local authority vending provider, using product lists available to the vender. It was vital that a ‘stepped change’ approach to delivering healthier solutions was adopted to drive this change but maintain a commercially viable level of sales throughout the process. This enabled successful collaboration with regional local authorities building on previous work in the city and following best practice approaches, in this case GBSF in England. Support is needed for local policy teams to work with businesses to put new specifications in place. The addition of ITS analysis strengthened the analysis and confirmed that underlying trends (due to factors external to the intervention) were minimal in terms of energy and nutrients, further supporting the results obtained from the pre-post regression analysis.

There were notable limitations related to this study such as the lack of a control group. The use of a sample of similar vending machines in similar locations as a control group where no changes were made would have further strengthened the analysis. In addition, the length of time for each phase varied substantially and was generally short in order to fit in with local authority and provider intervention timelines, which made evaluation more challenging. Although the baseline phase was one of the shorter phases, it did, however, meet the minimum standards necessary for robust ITS analysis of eight data points^(32)^. The number of machines chosen was a decision based on availability of machines provided by the vending company and not based on power calculations, although it is likely that the sample was sufficient to identify moderate improvements in availability and purchasing based on retrospective power calculations. Additionally, there is a risk of bias if the included machines provided by the vending company differed from suppliers of other vending machines in English sports centres. There were some difficulties in phase 2 to find suitable products despite using the full list of products available to the supplier. Key sellers, such as cookies, were challenging to replace with like-for-like products, potentially leading to customers switching to other categories. Recent further extensions of the GBSF nutrition criteria to additional products^(17)^ may lead to further reformulation and wider availability of smaller portion sizes of snack foods from food manufacturers.

Design of future interventions should consider that multicomponent interventions that include marketing, pricing and access strategies rather than solely focusing on availability may be even more effective in encouraging healthier vending purchases. Care needs to be taken to include food environment components and not just educational-based strategies that may be more prone to increase inequalities in dietary behaviour^(33)^. In terms of evaluating interventions, universally agreed methods of assessment of quality of vending machine provision are needed. In a review of vending machine assessment^(20)^, the authors noted that measures evaluating vending machine interventions are highly inconsistent. Nevertheless, the outcomes chosen here, which included nutrients purchased, are common outcomes, and this work on improving the nutrient profile of snack products in vending machines can potentially inform future interventions. Future evaluation work could consider reporting of nutrients beneficial to the diet, such as fibre, instead of solely focusing on reduction of fats, sugars and salt, as previously highlighted^(20)^. Future policy steps could also include regional commitments to improve the public sector food environment. Ultimately, national changes in the nutritional profile of vended snacks in combination with other changes to the food environment could help to shift the population towards healthier dietary intakes.
